# Dental Professionals’ Knowledge of Oral Health in Chronic Kidney Disease: Exploratory Survey

**DOI:** 10.1016/j.identj.2026.109815

**Published:** 2026-08-17

**Authors:** Reinhard Chun Wang Chau, Prakash Gudsoorkar, Isabella Lerma, Sompop Bencharit, Fernando Neves Hugo, Abhijit V. Kshirsagar, Edgar Lerma, Ryan Lee, Daniel Croley, Sujay A.J. Mehta, Sabine Karam, Lakshman Samaranayake, Priyanka Gudsoorkar

**Affiliations:** a Department of International and Community Oral Health, Graduate School of Dentistry, Tohoku University, Sendai, Japan; b Faculty of Dentistry, The University of Hong Kong, Hong Kong SAR, China; c Division of Nephrology, Department of Internal Medicine, University of Cincinnati, Cincinnati, Ohio, USA; d Marquette University, Milwaukee, Wisconsin, USA; e Austin Institute of Dental Medicine, Austin, Texas, USA; f Digital Dentistry & Innovation LLC, Chapel Hill, North Carolina, USA; g Department of Restorative Sciences, Adams School of Dentistry, University of North Carolina at Chapel Hill, Chapel Hill, North Carolina, USA; h Department of Epidemiology and Health Promotion, New York University College of Dentistry, New York, New York, USA; i Division of Nephrology and Hypertension and UNC Kidney Center, University of North Carolina, Chapel Hill, North Carolina, USA; j Department of Medicine, Section of Nephrology, University of Illinois at Chicago, Chicago, Illinois, USA; k Associates in Nephrology, Chicago, Illinois, USA; l US Army Reserve Command, Fort Liberty, North Carolina, USA; m Caring Health Center, Springfield, Massachusetts, USA; n Delta Dental of California, San Francisco, California, USA; o Department of Medicine, Faculty of Health Sciences, McMaster University, Hamilton, Ontario, Canada; p Division of Nephrology and Hypertension, Department of Medicine, University of Minnesota, Minneapolis, Minnesota, USA; q Division of Nephrology and Hypertension, Department of Internal Medicine, American University of Beirut, Beirut, Lebanon; r Global Research Cell, Dr. D. Y. Patil Dental College and Hospital, Dr. D. Y. Patil Vidyapeeth, Pimpri, Pune, India; s Department of Environmental & Public Health Sciences, College of Medicine, University of Cincinnati, Cincinnati, Ohio, USA

**Keywords:** Renal insufficiency Chronic, Oral health, Periodontitis, Interdisciplinary communication, Education Dental, Surveys and questionnaires

## Abstract

**Introduction and aims:**

The bidirectional relationship between periodontitis and chronic kidney disease (CKD) underscores the importance of oral health in CKD management. However, interdisciplinary collaboration between dental and nephrology professionals remains limited. This study assessed dental professionals’ knowledge, clinical experiences, referral patterns, perceived barriers, and attitudes towards integrated oral-renal care.

**Methods:**

A cross-sectional web-based survey was conducted internationally in Asia, North America, Africa, Europe, South America, and Australia from November 2025 to February 2026. The instrument explored demographics, clinical exposure to CKD/end-stage kidney disease patients, self-assessed knowledge and confidence, referral/communication patterns, barriers to coordination, and interest in interdisciplinary protocols. Data from 89 valid responses were analysed using descriptive and inferential statistical methods.

**Results:**

Respondents were predominantly experienced practitioners (74.2% with >10 years of practice; 67.4% had treated CKD patients; 53.9% dialysis patients). The self-assessed composite knowledge-and-confidence score for oral-renal interactions was moderate (mean 7.67/12). Referrals from nephrologists and direct interdisciplinary communication were infrequent. Primary barriers included a lack of a clear point of contact (51.7%) and a lack of shared patient records (50.6%). Oral health was rated as very or moderately important in CKD management by 94.4% of respondents, and 100% expressed definite or possible interest in formal interdisciplinary care protocols.

**Conclusions:**

Despite moderate self-assessed knowledge and substantial clinical exposure, structural and systems-level barriers appear to impede the integration of oral-renal care. Dental professionals nevertheless demonstrate near-universal motivation for interdisciplinary collaboration and strong interest in formal care protocols.

**Clinical relevance:**

Findings highlight the need for educational initiatives, standardized referral pathways, interoperable records, and policy support to advance multidisciplinary care for CKD patients.

## Introduction

The oral cavity occupies a unique position at the interface of local and systemic health. Dental providers increasingly encounter patients with complex medical histories, and the recognition that oral disease both reflects and perpetuates systemic pathology has fundamentally reshaped contemporary dental practice. Among the systemic conditions of great clinical relevance to the dental team, chronic kidney disease (CKD) and its end-stage manifestation, end-stage kidney disease (ESKD), represent a growing imperative for the dental team. The bidirectional relationship between periodontitis and CKD is now well established. Periodontitis, a dysbiosis-driven chronic inflammatory disease affecting approximately one billion people in its severe form, is recognized as a modifiable risk factor for CKD and other noncommunicable diseases.^1^ Ulceration of the periodontal pocket epithelium allows translocation of periodontal pathogens, lipopolysaccharide, and proinflammatory mediators (IL-6, TNF-α, CRP) into the circulation, amplifying the systemic inflammatory burden characteristic of CKD.^1^^,^^2^ In turn, the uremic environment of CKD exacerbates periodontal tissue destruction through immune dysregulation, neutrophil dysfunction, and reduced salivary flow, creating a self-reinforcing pathogenic cycle.^3^^,^^4^ Systematic reviews and meta-analyses consistently show that periodontitis is associated with increased risk of CKD incidence and progression, declining eGFR, and elevated inflammatory markers.^1^^,^^4^, ^5^, ^6^, ^7^, ^8^ Nonsurgical periodontal therapy has been shown to reduce CRP and IL-6 levels in CKD populations, suggesting that periodontal care may serve as a modifiable upstream intervention to reduce systemic inflammatory load.^4^^,^^9^, ^10^, ^11^, ^12^, ^13^ Previous studies noted that oral health manifestations in CKD were active drivers of systemic inflammatory burden, infection risk, protein-energy wasting, and reduced quality of life (outcomes that are directly amenable to dental intervention), and called for routine oral assessments and structured cross-specialty collaboration as a necessary component of comprehensive CKD management.^13^^,^^14^

CKD currently affects an estimated 697.5 million individuals worldwide, with a global prevalence approaching 9.1%, and its burden is rising inexorably alongside the global epidemics of diabetes mellitus and hypertension, its two principal etiological drivers.^14^^,^^15^ Analysis of Global Burden of Disease data from 1990 to 2016 further demonstrates that 63% of the CKD burden is concentrated in low- and lower-middle-income countries, where late diagnosis and limited access to specialist care compound disease progression.^16^

For dental clinicians, these epidemiological trends carry direct clinical consequences. These include xerostomia and salivary hypofunction, reported in 28% to 79% of ESKD patients, as well as uremic stomatitis, mucosal pallor, dysgeusia, gingival bleeding diathesis secondary to platelet dysfunction, and accelerated dental caries.^17^, ^18^, ^19^ In patients receiving renal replacement therapy, the dental management context is further complicated by the need to schedule invasive procedures appropriately around dialysis sessions, account for heparin-mediated anticoagulation, and consider antibiotic prophylaxis protocols, particularly for patients receiving peritoneal dialysis, in whom invasive dental procedures may precipitate peritonitis.^20^, ^21^, ^22^, ^23^ Patients awaiting kidney transplantation require comprehensive pretransplant dental clearance, as post-transplant immunosuppression substantially amplifies susceptibility to both local and systemic infectious complications arising from untreated oral disease.^24^, ^25^, ^26^, ^27^, ^28^, ^29^, ^30^ These clinical imperatives establish the dental team as integral contributors to the multidisciplinary management of CKD across its clinical continuum.

Despite the growing body of evidence linking oral and renal health and the calls for coordinated, synergistic care, the translation of this knowledge into multidisciplinary clinical practice remains substantially incomplete. Structural barriers, including divergent educational pathways, separate healthcare financing systems, and a lack of interoperability between dental and medical electronic health records, perpetuate the functional disconnect between dental and nephrology care.^9^^,^^31^ Communication between dental professionals and nephrologists is predominantly informal and reactive, driven by individual provider initiative rather than by standardized referral pathways, resulting in delayed or absent interdisciplinary engagement.^9^

Compounding this fragmentation, current clinical practice guidelines from major nephrology bodies (eg, KDIGO) and the key representative periodontal professional organizations (eg, American Academy of Periodontology, European Federation of Periodontology) do not provide dedicated, coordinated recommendations for periodontal or dental care provision in patients with CKD or ESKD.^32^, ^33^, ^34^ The absence of authoritative, society-endorsed recommendations leaves individual clinicians without a standardized reference framework. It removes a key lever through which payers and health systems typically recognize and reimburse coordinated care.^9^

This fragmentation has led the American Society of Nephrology (ASN) to submit a formal 2025 policy proposal to the Centers for Medicare & Medicaid Services advocating for Medicare coverage of medically necessary dental care for patients with diabetes and CKD.^35^ The 2026 Medicare Physician Fee Schedule has also introduced integration-linked incentives. Yet, despite these developments, comprehensive dental coverage for CKD populations remains fragmented and inconsistent, and meaningful nephrology-dentistry collaboration has not yet been systematically established.^9^

Against this background, the perspectives of dental professionals regarding their experience with CKD and ESKD patients, familiarity with relevant oral-systemic interactions, referral behaviours, barriers to coordinating care, and views on how these barriers might be addressed remain incompletely characterized in the published literature. Understanding these perspectives is a prerequisite to the design of effective, evidence-based integration frameworks.

The objectives of this study were therefore:

(1) to describe the demographic and practice characteristics of dental professionals, their clinical exposure to CKD and ESKD patients (including dialysis and transplant populations), and their self-assessed knowledge, confidence, and familiarity with oral health complications and management considerations in this population;
(2) to characterize referral patterns, interdisciplinary communication with nephrologists, and perceived barriers to coordinated oral-renal care; and
(3) to evaluate attitudes towards the importance of oral health in CKD/ESKD management and interest in formal interdisciplinary care protocols.

As an exploratory cross-sectional survey, no prespecified hypotheses were formulated. The findings are intended to identify specific knowledge and practice gaps, inform the development of standardized interdisciplinary protocols, and contribute to the broader evidence base supporting integrated oral-renal care as a clinical and policy priority.

## Methods

*Study design and setting.* A cross-sectional, convenience-sampled, anonymous, web-based survey was conducted between November 2025 and February 2026 to assess dental professionals’ clinical experiences, knowledge, referral patterns, and attitudes towards interdisciplinary collaboration in managing patients with CKD and ESKD. The study was designed as an exploratory investigation to characterize current practices and identify structural, communication, time-resource, financing, and systems-level barriers to oral–renal care integration. The research protocol was approved by the Institutional Review Board at the University of Cincinnati (#2025-0227). Participation was entirely voluntary, and electronic informed consent was obtained from all respondents before survey initiation. No personally identifiable information was collected. The study was reported in accordance with the Strengthening the Reporting of Observational Studies in Epidemiology (STROBE) guidelines.^36^

*Sample size.* The study’s sample size was not predetermined through formal sample-size or power calculations. As an exploratory convenience-sampled survey, the final analytical sample was determined by the number of eligible responses received during the 4-month dissemination period.

*Survey instrument development.* The survey instrument was structured across six thematic domains, based on existing literature on oral–systemic health interactions in CKD populations and emerging frameworks for multidisciplinary chronic disease management^1^^,^^4^^,^^9^; (1) respondent demographics and practice characteristics; (2) clinical experience with CKD and ESKD patients, including dialysis and transplant populations; (3) self-assessed knowledge of oral health complications associated with CKD and familiarity with cardio-renal-metabolic (CRM) syndrome; (4) referral and interdisciplinary communication patterns; (5) perceived barriers to coordination with nephrology services; and (6) attitudes towards and interest in formal interdisciplinary care protocols. Items employed Likert-type rating scales, multiple-choice formats, and select-all-that-apply questions, with optional open-ended responses. The instrument was piloted among a small group of dental clinicians to optimize clarity and internal coherence prior to distribution. A composite knowledge-and-confidence score was constructed by summing three self-reported items:

1. confidence in managing medically complex patients with CKD/ESKD (1-5 scale),
2. familiarity with CRM syndrome and its relevance to oral health (1-4 scale), and
3. familiarity with antibiotic prophylaxis for dental treatment in patients on peritoneal dialysis (1-3 scale).

The resulting score ranged from 3 to 12, with higher scores indicating greater self-assessed knowledge and confidence. Internal consistency of this three-item composite was assessed using Cronbach’s alpha.

*Survey administration and dissemination.* The survey was built and hosted using Qualtrics XM^37^ and disseminated internationally through professional dental networks (American Dental Association, Oral Health Section of the American Public Health Association, IADR), digital platforms, targeted social media channels (LinkedIn), and purposive email outreach to academic and clinical collaborators. To broaden accessibility, French and Spanish versions were introduced in the second dissemination phase; translations were generated using the Google Translate API and reviewed by fluent speakers. reCAPTCHA v3 verification (score ≥0.5) was implemented to prevent automated or fraudulent submissions.

*Eligibility and response processing.* Dental professionals were eligible to participate regardless of practice setting, geographic location, or years of experience. Responses were excluded from the analytical dataset if the respondent explicitly withheld consent to be included or if the survey was incomplete. Of the (*n* = 148) total responses received, 60.14% (*n* = 89) fulfilled the eligibility criteria and were retained for analysis.

*Addressing potential sources of bias.* Convenience sampling through professional networks and social media channels carries an inherent risk of selection bias towards more engaged and digitally connected practitioners. To mitigate this and other potential biases (language, social desirability, and invalid responses), the survey was disseminated via multiple international channels, offered in three languages, kept fully anonymous with no financial incentives, pilot-tested, and protected by reCAPTCHA verification. Only complete responses with explicit consent were included in the analytical dataset. No poststratification weighting was applied, given the exploratory nature of the study.

*Statistical analysis.* Data extraction and initial cleaning were performed in Microsoft Excel.^38^ All statistical analyses were conducted using Python version 3.12.3, employing the Pandas, SciPy, Statsmodels, Pingouin, and PyMC libraries.^39^ Descriptive statistics are reported as frequencies (*n*) and percentages (%) for categorical variables, calculated to three decimal places. For multiselect items, proportions were computed relative to the total valid sample (*n* = 89), as multiple responses per respondent were permissible; accordingly, percentage totals for these items may exceed 100%. The distributional properties of key ordinal variables were assessed using the Shapiro–Wilk test, with *P* < .05 indicating a significant departure from normality and the appropriateness of nonparametric inferential procedures.

Inferential analyses included Chi-square tests and Fisher’s exact tests for the examination of associations between categorical variables, the Kruskal–Wallis test for between-group comparisons of ordinal data, Spearman’s rank correlation for bivariate associations among ordinal measures, multivariate logistic regression to identify variables associated with binary outcomes of interest, and sensitivity analyses to assess the robustness of primary findings. Given the nonprobability convenience sampling strategy, sampling weights, design-based variance estimation, or complex-survey adjustments were not applied, and all analyses were performed on the unweighted analytical sample. Cronbach’s alpha was calculated to evaluate the internal consistency of the three-item composite knowledge-and-confidence score.

Qualitative Analysis of Open-Ended Responses. Open-ended responses were analysed using descriptive content analysis. Non-English submissions were translated into English before coding. Thematic categories were derived inductively through iterative review of the response corpus, with recurring concepts organized into substantive themes encompassing referral infrastructure, communication limitations, educational needs, and systemic access barriers. Theme frequencies were quantified and reported after the final coding round.

## Results

**Fig. 1:**
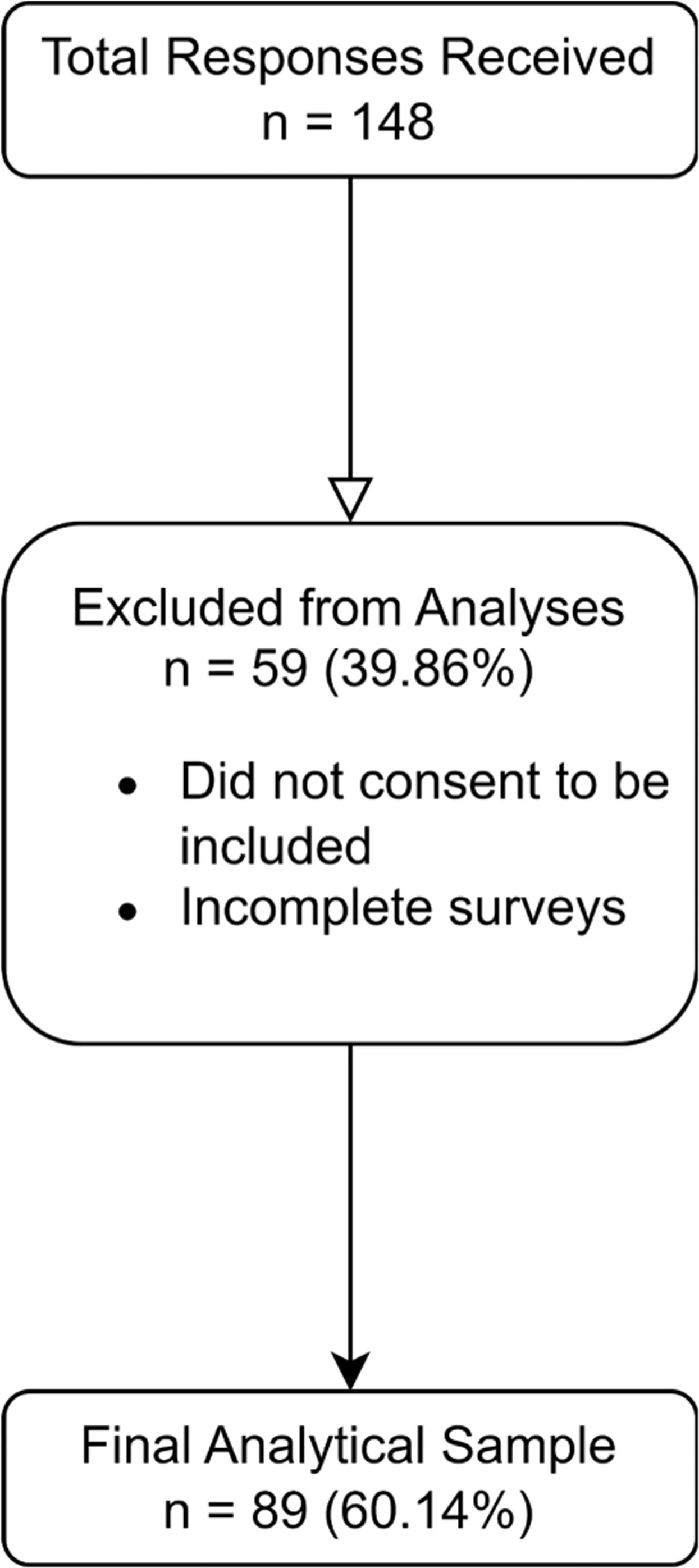
Flow of participants through the cross-sectional survey.

Of 148 total responses received during the dissemination period, 59 were excluded because respondents did not provide consent or submitted incomplete surveys. The final analytical sample comprised 89 eligible dental professionals (60.14%) (Figure 1).

The analytical sample (*n* = 89) was heavily skewed towards senior practitioners: collectively, 74.16% (*n* = 66) had accumulated more than a decade of dental practice, with nearly half reporting more than 20 years (*n* = 43, 48.32%) and a further 25.84% (*n* = 23) reporting 11 to 20 years; the remaining respondents comprised those with 5 to 10 years of practice (*n* = 9, 10.11%) and fewer than 5 years (*n* = 14, 15.73%). This seniority was reflected in the age distribution, with 32.58% (*n* = 29) aged 46 to 55 years, the largest single age group, and 57.30% (*n* = 51) of the study sample aged 46 years or older. Consistent with this experience profile, most respondents reported substantive prior clinical exposure to patients with kidney disease: 67.42% (*n* = 60) had provided dental treatment to a patient with CKD, and more than half, 53.93% (*n* = 48), had managed a patient currently receiving haemodialysis or peritoneal dialysis.

Taken together, these characteristics establish the sample as a clinically experienced sample of dentists whose responses reflect accumulated direct exposure to this patient population. Full demographic frequencies and clinical exposure data are presented in Table 1, Table 2, respectively.

**Table 1:** Demographic characteristics of valid survey respondents (*n* = 89).

Demographic variable	Category	*n* (%)
Age group	20-35 y	17 (19.10%)
	36-45 y	23 (25.84%)
	46-55 y	29 (32.58%)
	56-65 y	12 (13.48%)
	65+ y	8 (8.99%)
Years of experience in dental practice	Less than 5 y	14 (15.73%)
	5-10 y	9 (10.11%)
	11-20 y	23 (25.84%)
	More than 20 y	43 (48.32%)
Geographic region	Asia	33 (37.08%)
	North America	26 (29.21%)
	Africa	13 (14.61%)
	Europe	8 (8.99%)
	South America	8 (8.99%)
	Australia/New Zealand	1 (1.12%)
Primary practice setting	Academic medical centre	34 (38.20%)
	Private practice	31 (34.83%)
	Public/government facility	21 (23.60%)
	Other	3 (3.37%)

Data are reported as frequencies (*n*) and percentages (%). Percentage sums within each variable may not equal 100% due to rounding.

**Table 2:** Clinical experience with patients with kidney disease among valid respondents (*n* = 89).

Survey item	Response	*n*	%
Have you ever provided dental treatment to a patient with chronic kidney disease (CKD)?	Yes	60	67.42
	No/not sure	29	32.58
Have you ever provided dental care for a patient currently on dialysis (haemodialysis or peritoneal dialysis)?	Yes	48	53.93
	No/not sure	41	46.07

Data are reported as frequencies (*n*) and percentages (%). ‘No/not sure’ responses were collapsed for analysis as both represent the absence of confirmed clinical exposure. Percentage sums within each item may not equal 100% due to rounding.

The sample was diverse geographically, spanning six global regions (Table 1; Figure 1). The largest proportions of respondents were based in Asia (*n* = 33, 37.08%) and North America (*n* = 26, 29.21%). Notably, more than one-quarter of the cohort represented regions with Africa (*n* = 13, 14.61%), South America (*n* = 8, 8.99%), and Europe (*n* = 8, 8.99%), with minimal representation from Australia/New Zealand (*n* = 1, 1.12%). With respect to primary practice setting, the largest proportion of respondents were based in academic medical centres (*n* = 34, 38.20%), followed by private practice (*n* = 31, 34.83%), public or government facilities (*n* = 21, 23.60%), and other settings (*n* = 3, 3.37%; Table 1).

**Fig. 2:**
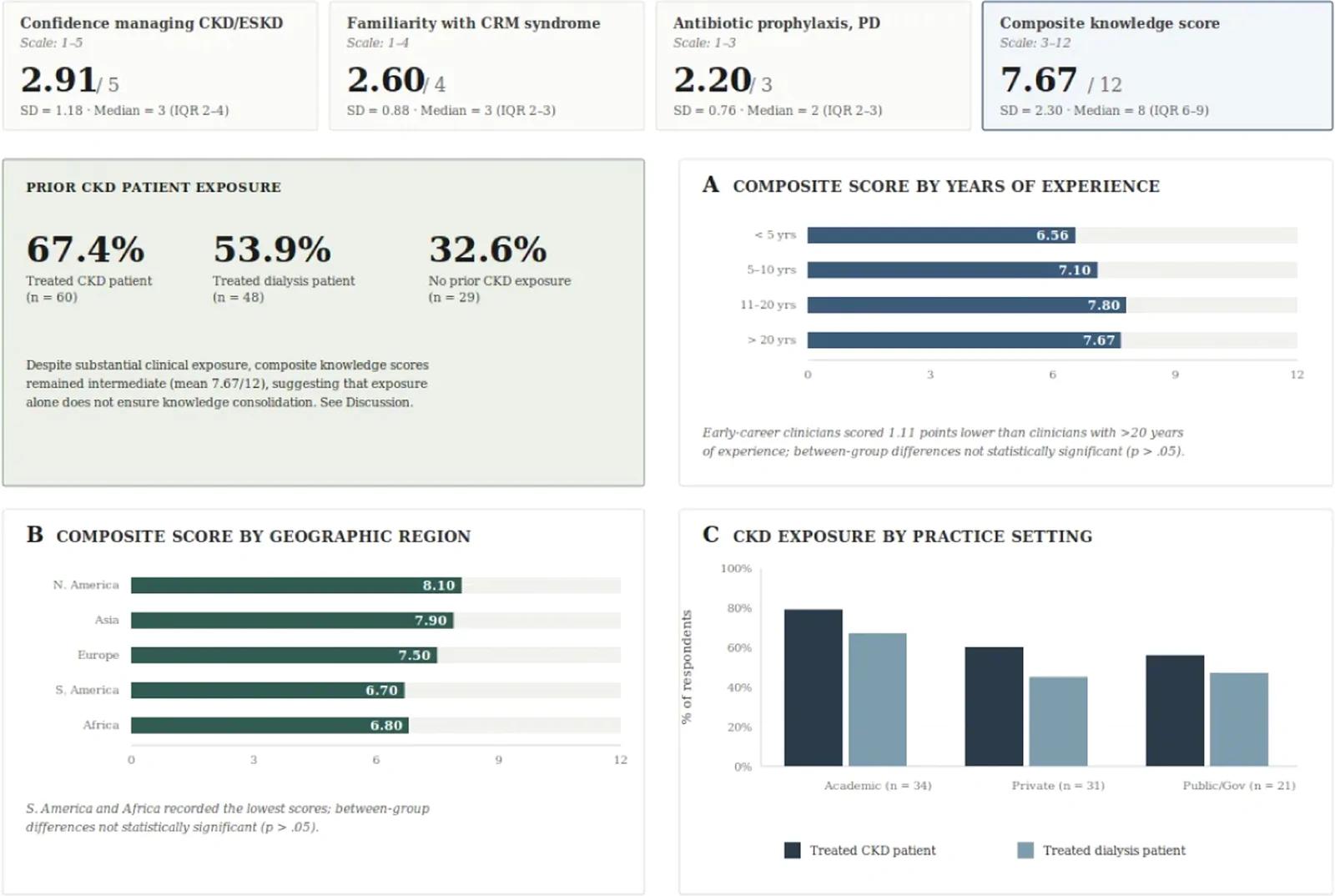
Self-assessed knowledge, confidence, and prior clinical experience with CKD/ESKD patients (*n* = 89). *Note.* Upper metric cards display the mean (SD) and median (IQR) for each knowledge domain on its respective scale; the composite knowledge-and-confidence score (range 3-12), prior clinical exposure to CKD and dialysis patients, and the knowledge discordance observation. Parts (A and B) illustrate composite knowledge-and-confidence score stratified by years of clinical experience and geographic region, respectively. Part (C) displays the proportion of respondents who had previously treated patients with CKD or on dialysis, stratified by primary practice setting.

Self-assessed confidence in managing medically complex patients with CKD or ESKD, rated on a scale of 1 (not at all confident) to 5 (very confident), yielded a mean score of 2.91 (SD = 1.18), with a median of 3 (IQR = 2-4), indicating a moderate and heterogeneous level of confidence across the cohort. Familiarity with CRM syndrome and its relevance to oral health, assessed on a scale of 1 (not familiar) to 4 (very familiar), yielded a mean score of 2.60 (SD = 0.88), with a median of 3 (IQR = 2-3). Familiarity with antibiotic prophylaxis for dental treatment in patients receiving peritoneal dialysis, rated on a scale of 1 (not familiar) to 3 (very familiar), had a mean score of 2.20 (SD = 0.76) and a median of 2 (IQR = 2-3). The composite knowledge-and-confidence score, derived from the summation of all three domains on a scale of 3 to 12, yielded a mean of 7.67 (SD = 2.30) and a median of 8 (IQR = 6-9), with an acceptable internal consistency of the three-item scale (Cronbach’s *α* = 0.70). Shapiro–Wilk tests confirmed that none of the knowledge-related scales were normally distributed (all *P* < .05), supporting the use of nonparametric approaches for subsequent inferential analyses. Domain-level and composite knowledge-and-confidence scores are summarized and stratified in Figure 2.

Stratification of composite knowledge-and-confidence score by years of clinical experience revealed a positive gradient, with scores increasing as professional seniority increased, though the difference was not statistically significant (*H* = 4.35, *P* = .23, *ε*² = 0.02). Early-career clinicians scored, on average, 1.11 points lower than those with more than 20 years of practice. However, this difference did not attain statistical significance (*P* > .05). Spearman’s rank correlation also confirmed no statistically significant association between years of experience and the knowledge-and-confidence score (*ρ* = 0.20, *P* = .06). The difference in composite knowledge-and-confidence scores by geographic region did not reach statistical significance (*H* = 7.20, *P* = .21, *ε*² = 0.03). Still, respondents from South America (mean = 6.38) and Africa (mean = 6.92) recorded relatively lower mean scores.

Patterns of referral and interdisciplinary communication were markedly limited across the cohort. Many respondents had never received a referral from a nephrologist (*n* = 56, 62.92%), and 70.79% (*n* = 63) reported no confirmed direct communication with a nephrologist regarding a shared patient. Referral frequency was examined across the three principal practice settings and is reported in full in Table 3. Among respondents in private practice (*n* = 31), 70.97% (*n* = 22) reported zero referrals per month, 22.58% (*n* = 7) reported approximately one referral per month, and 6.45% (*n* = 2) reported two to five referrals per month. Among respondents based in academic medical centres (*n* = 34), 52.94% (*n* = 18) reported no monthly referrals, 26.47% (*n* = 9) reported one per month, 17.65% (*n* = 6) reported two to five per month, and 2.94% (*n* = 1) reported more than 10 per month; this was the only respondent across any practice setting to do so. Among respondents in public or government facilities (*n* = 21), 71.43% (*n* = 15) reported zero referrals per month, 19.05% (*n* = 4) reported one per month, and 9.52% (*n* = 2) reported two to five per month. Chi-square analysis revealed no statistically significant variation in referral frequency across the three settings (*χ*² = 11.23, *P* = .08, Cramér’s *V* = 0.26), though a trend towards higher referral volumes among academic centre respondents was observed, consistent with the structural advantage of colocated specialist services (Table 3).

**Table 3:** Referral frequency by primary practice setting (*n* = 86).

Primary practice setting	None *n* (%)	1 per mo *n* (%)	2-5 per mo *n* (%)	>10 per mo *n* (%)	Distribution
Setting (total *n*)					
Private practice *n* = 31	22 (70.97)	7 (22.58)	2 (6.45)	0 (0.00)	71% 23% 6%
Academic medical centre *n* = 34	18 (52.94)	9 (26.47)	6 (17.65)	1 (2.94)	53% 26% 18%
Public/government facility *n* = 21	15 (71.43)	4 (19.05)	2 (9.52)	0 (0.00)	71% 19% 10%
**Total**	**55**	**20**	**10**	**1**	

Three respondents reporting ‘other’ as their primary practice setting were excluded, yielding an analytical sample of *n* = 86. Row percentages are calculated within each practice setting.

**Fig. 3:**
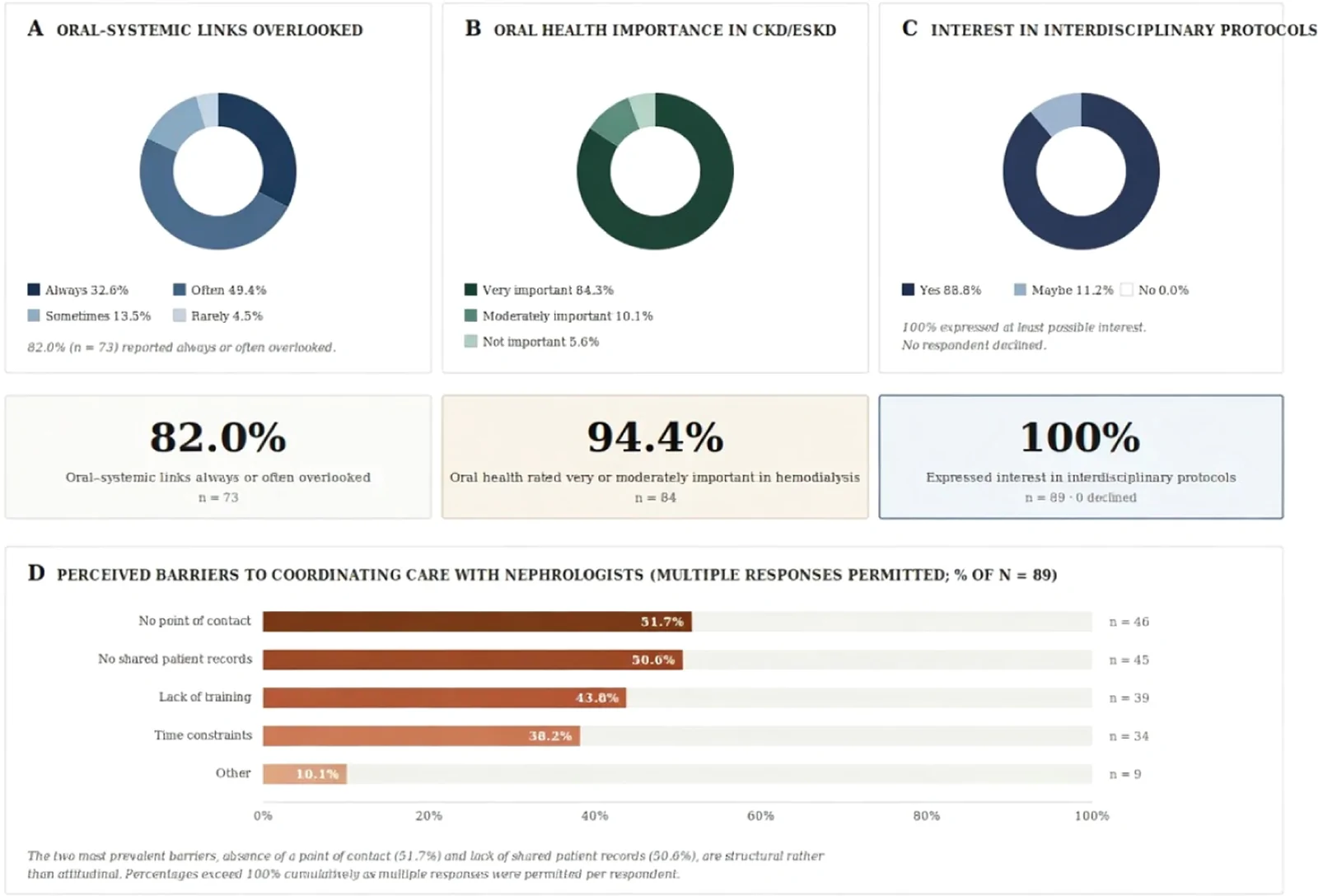
Barriers to interdisciplinary coordination, attitudes towards oral–systemic integration, and interest in care protocols (*n* = 89). (A) Frequency with which respondents perceived oral–systemic connections to be overlooked in interdisciplinary care. (B) Perceived importance of oral health in the overall management of CKD and ESKD. (C) Level of interest in the development of formal interdisciplinary care protocols…

The barriers underlying this limited interdisciplinary engagement, together with respondents’ attitudes towards oral–systemic integration and interest in formal protocols, are illustrated collectively in Figure 3. When asked to identify obstacles to coordinating care with nephrologists (with multiple responses permitted) the two most frequently endorsed barriers were the absence of a clear point of contact (*n* = 46, 51.69%) and the lack of shared patient records (*n* = 45, 50.56%), followed by lack of training (*n* = 39, 43.82%), time constraints (*n* = 34, 38.20%), and other unspecified barriers (*n* = 9, 10.11%; Figure 3D). The predominance of structural and infrastructural barriers is of clinical and policy significance, suggesting that the deficit in collaboration is driven primarily by system-level deficiencies rather than by professional disengagement.

Against this backdrop of limited coordination, respondents expressed a striking degree of consensus regarding the importance of oral–systemic integration (Figure 3). The overwhelming majority perceived oral–systemic connections to be consistently overlooked in interdisciplinary care: 32.58% (*n* = 29) reported this occurs always, and 49.44% (*n* = 44) reported it occurs often, together constituting 82.02% (*n* = 73) of the valid sample (Figure 3A). Only 4.49% (*n* = 4) indicated that such connections are rarely overlooked, and no respondent indicated they are never overlooked. With respect to the perceived importance of oral health in the overall management of CKD and ESKD, 84.27% (*n* = 75) rated it as very important and a further 10.11% (*n* = 9) as moderately important, yielding a combined affirmative response rate of 94.38% (*n* = 84; Figure 3B).

The most widely endorsed finding of the survey was near-universal interest in formal interdisciplinary care protocols: 88.76% (*n* = 79) expressed definite interest, and 11.24% (*n* = 10) indicated possible interest, with no respondents indicating a lack of interest (Figure 3C). Critically, this motivation for integration was uniformly distributed across the cohort. Chi-square analyses demonstrated no statistically significant associations between interest in interdisciplinary protocols and age group, years in practice, or prior experience treating a CKD patient (all *P* > .30), and logistic regression modelling confirmed that no demographic or experiential variable significantly predicted interest (*P* = .68). Since 100% of respondents indicated definite or possible interest, there was no variance, and thus a sensitivity analysis was not performed. The finding that enthusiasm for collaboration is both high and independent of professional background constitutes a clear foundational condition for implementing structured, evidence-based oral–renal care guidelines.

## Discussion

This international survey identifies a gap between the recognized relevance of oral–renal interactions and their integration into routine dental practice. Despite a cohort weighted towards senior practitioners, self-assessed knowledge and confidence regarding CKD- and ESKD-related dental management remained only moderate, suggesting that clinical exposure alone does not ensure preparedness to manage this population safely. This is notable given the complexity of CKD and ESKD care, including oral manifestations, altered pharmacokinetics, haemostatic disturbances, and procedure-specific risks such as peritonitis associated with invasive dental treatment in patients receiving peritoneal dialysis.^4^^,^^20^^,^^21^ Taken together, these findings support the need for stronger integration of oral-systemic medicine within dental education and practice.^9^^,^^40^^,^^41^

Regional variation in knowledge scores adds an important equity dimension to these findings. Respondents from South America and Africa had the lowest mean composite knowledge-and-confidence score, a pattern consistent with structural disparities in access to continuing professional education in lower-resource settings. This is especially relevant because 63% of the global CKD burden is concentrated in low- and lower-middle-income countries,^16^ where the need for informed interdisciplinary dental care is substantial. Addressing this gap will require educational and technological strategies that are scalable, globally accessible, and not limited to high-resource academic environments.^42^, ^43^, ^44^, ^45^, ^46^, ^47^

The findings also suggest that predoctoral training alone may be insufficient to equip dental professionals for the safe management of medically complex patients, including those with CKD and ESKD. The foundational dental curriculum often provides limited preparation in interprofessional care, chronic disease management, and the medical decision-making required for patients with significant systemic illness. Where available, postgraduate hospital dentistry or medically focused residency training may offer a more appropriate environment for developing these competencies. At the same time, the strong interest in interdisciplinary collaboration observed across this cohort indicates that educational reform should be paired with broader systems-level change.

A central finding of this study is that the major barriers to oral–renal integration appear to be structural rather than attitudinal. These findings suggest that dental professionals are willing to collaborate but often lack the mechanisms to do so. However, the cross-sectional survey design precludes definitive causal conclusions about the primacy of structural barriers. The near-absence of formal referral pathways and direct communication with nephrologists, together with the frequent identification of missing points of contact and lack of shared patient records, indicates that dental professionals are willing to collaborate but often lack the mechanisms to do so. This distinction has important implications for intervention design. Efforts focused solely on clinician education are unlikely to be sufficient unless they are accompanied by referral infrastructure, interoperable records, and defined cross-specialty communication channels.^9^^,^^31^ Although not statistically significant, referral volumes were notably higher in institutional settings, possibly reflecting colocation and institutional integration.

These findings emerge within a shifting policy context. The ASN’s 2025 policy submission advocating Medicare coverage of medically necessary dental care for patients with CKD, together with integration-linked incentives in the 2026 Medicare Physician Fee Schedule, suggests a regulatory environment that is increasing policy attention to formalized oral–renal coordination.^9^^,^^35^^,^^48^

Financing and implementation remain critical and underexamined issues in oral–renal integration. Even when professional motivation and educational content are present, sustained change depends on reimbursement structures that support team-based, preventive, and chronic-care coordination across the oral–medical interface, as well as the operational infrastructure needed to support such care. In the United States, dental services remain largely excluded from medical insurance and are often paid out of pocket for adult Medicare beneficiaries. In many low- and middle-income countries, public dental financing is similarly fragmented or limited.

The ASN’s 2025 advocacy represents an important opening, but broader alignment from organized dentistry and from health ministries outside the United States will likely be necessary to support coordinated care on both sides of the interface. Although integrated medical–dental models have been described, including colocated care, community health centre integration, and chronic-care approaches that incorporate oral health, their implementation and cost-effectiveness for CKD-specific pathways remain insufficiently characterized, and direct evidence of improved renal or oral–renal outcomes remains limited. Translating professional readiness into sustained practice change will therefore require coordinated investment in clinical guidelines, financing reform, and pragmatic implementation research.

Several limitations of the study should be considered. The cross-sectional design precludes causal inference, and all knowledge, confidence, and familiarity measures were self-reported, introducing the possibility of social desirability and reporting bias. Self-perceived competence might not accurately reflect objective clinical knowledge or actual practice behaviours. The survey instrument was piloted for clarity and validity with a small group of dental professionals but did not undergo formal psychometric validation. As a result, the composite knowledge-and-confidence score, while useful for exploratory purposes, had unknown psychometric properties beyond the exploratory Cronbach’s *α* (0.70), and conclusions drawn from it should be interpreted with caution. The sample size was modest, and recruitment through professional networks and social media may have favoured more engaged and digitally connected clinicians. Certain regions, particularly Australia/New Zealand and South America, were underrepresented, precluding robust regional comparisons and limiting insights into potential cultural or healthcare-system influences on responses. The overrepresentation of clinicians with more than a decade of practice may limit the generalizability to early-career dental professionals.

Future research should build on these findings through larger, multicentre studies using stratified sampling, objective knowledge measures, and longitudinal designs. Interventional work should evaluate educational models that strengthen interprofessional competency in oral–systemic medicine in both predoctoral and postgraduate settings. Immersive dental education technologies may offer scalable training in managing oral disease, haemostatic risk, altered pharmacokinetics, and procedure-specific complications in CKD and ESKD.^49^^,^^50^ In parallel, mHealth tools and emerging artificial intelligence-enabled approaches may support real-time recognition of oral manifestations, risk stratification, and referral decision-making,^50^^,^^51^ particularly in lower-resource settings where the global CKD burden is concentrated. Implementation studies should also evaluate codesigned referral protocols, interoperable records, and cross-specialty liaison models to determine how clinician willingness can be translated into measurable improvements in coordinated care.^52^

## Conclusion

This survey suggests that dental professionals across diverse international practice contexts possess genuine motivation for interdisciplinary engagement with nephrology but operate within systems that structurally inhibit it. The principal barriers appear to be structural and financial rather than motivational. The near-universal interest in formal interdisciplinary care protocols indicates readiness for system-level solutions. Future work should focus on developing and testing practical referral pathways, shared-record solutions, and concise educational modules that can be implemented across practice settings.

## Author contributions

*Conceptualization*: Chau, Prakash Gudsoorkar, I. Lerma, Bencharit, Hugo, Kshirsagar, E. Lerma, Lee, Croley, Mehta, Karam, Priyanka Gudsoorkar. *Methodology*: Prakash Gudsoorkar, Bencharit, Hugo, Kshirsagar, E. Lerma, Lee, Croley, Mehta, Karam, Priyanka Gudsoorkar. *Software*: Chau, Priyanka Gudsoorkar. *Validation*: Chau, Priyanka Gudsoorkar. *Formal analysis*: Chau, Priyanka Gudsoorkar. *Investigation*: Chau, Prakash Gudsoorkar, I. Lerma, Bencharit, Hugo, Kshirsagar, E. Lerma, Lee, Croley, Mehta, Karam, Priyanka Gudsoorkar. *Resources*: Prakash Gudsoorkar, Bencharit, Hugo, Kshirsagar, E. Lerma, Lee, Croley, Mehta, Karam, Priyanka Gudsoorkar. *Data curation*: Chau, Priyanka Gudsoorkar. *Writing – original draft*: Chau, Prakash Gudsoorkar, I. Lerma, Priyanka Gudsoorkar. *Writing – review and editing*: Chau, Prakash Gudsoorkar, I. Lerma, Bencharit, Hugo, Kshirsagar, E. Lerma, Lee, Croley, Mehta, Karam, Priyanka Gudsoorkar. *Visualization*: Chau, Priyanka Gudsoorkar. *Supervision*: Prakash Gudsoorkar, Karam, Priyanka Gudsoorkar. *Project administration*: Prakash Gudsoorkar, Karam, Priyanka Gudsoorkar.

## Conflict of interest

The authors declare that they have no known competing financial interests or personal relationships that could have influenced the work reported in this article.

## Appendix Supplementary materials

Image, application 1
